# Bowel intussusception and adhesive intestinal obstruction in neurofibromatosis type 1

**DOI:** 10.1002/jgf2.547

**Published:** 2022-04-11

**Authors:** Tsunetaka Kijima, Shuko Ishida, Takashi Kishi, Minekazu Yamaguchi

**Affiliations:** ^1^ Oda Municipal Hospital, Oda Training Center of General Practice Faculty of Medicine, Shimane University Oda city Japan; ^2^ Department of Internal Medicine, Ohda Municipal Hospital Oda city Japan; ^3^ Department of Surgery, Ohda Municipal Hospital Oda city Japan

**Keywords:** adhesive intestinal obstruction, bowel intussusception, neurofibromatosis type 1

## Abstract

We report the case that a patient with Neurofibromatosis type 1 experienced bowel intussusception and adhesive intestinal obstruction. Bowel intussusception was considered to be due to long intestinal tube and multiple intraabdominal lesions including gastrointestinal stromal tumors (GISTs).

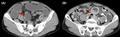

A 63‐year‐old man with neurofibromatosis type 1 (NF1) presented to the emergency department with a loss of appetite and vomiting for 3 days. He had abdominal pain 3 days prior to presentation, which started after consumption of partially cooked fish. Although the pain subsided, he was unable to eat. The patient had no other medical history, including previous abdominal surgery. His vital signs were normal. Abdominal examination revealed minor distention and no tenderness. Several neurofibromas and café au lait macules were observed. Computed tomography (CT) revealed an obstruction in the small intestine, suggesting an adhesion or cord‐like structure, which changed the caliber of the small intestine (Figure 1A). Bowel rest was instituted with long intestinal tube (long tube) drainage and intravenous fluid resuscitation. No significant change in the drainage volume was noted 4 days later. Gastrografin enterography revealed severe stenosis in the area of the adhesion and caliber change; however, complete obstruction was not observed. One week after hospitalization, contrast‐enhanced abdominal CT performed owing to persistently high drainage volume in the long tube revealed small bowel intussusception (Figure 1B). The cause of the adhesion was not evident. Although the patient did not complain of abdominal pain, surgery was performed the same day because long tube drainage had not relieved the obstruction; further, the long tube was suspected to cause the intussusception. We considered that the prolonged use of the long tube might increase the risk of ischemia in the bowel. During surgery, adhesions were discovered between the greater omentum and mesentery, invaginating the jejunum into a space bound by these tissues. Open surgical adhesiolysis was performed, and a small nodular fibrous adhesion and part of the greater omentum were resected (Figure 2A). The portion of the small intestine suspected of having small bowel intussusception appeared normal on a repeat contrast‐enhanced abdominal CT, and there were no ischemic changes noted intraoperatively. Moreover, two small nodules were identified at the serosal surface of the jejunum, 170 cm from the ligament of Treitz. These were resected along with the surrounding small intestine because they were suspected of causing the intussusception (Figure 2B). These nodules were histopathologically found to be gastrointestinal stromal tumors (GISTs). No other intraluminal tumors were palpable intraoperatively. A small nodule approximately 5 mm from the ligament of Treitz was not resected because it was considered unlikely to cause symptoms. We retrospectively found that the GISTs were not visible on the preoperative enhanced CT. The patient's abdominal distention resolved, and he started consuming food orally 5 days postoperatively. He was discharged from the hospital 6 days later.

**FIGURE 1:**
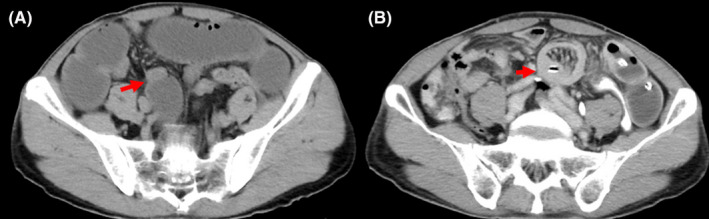
(A) Abdominal CT showing an adhesion or cord‐like structure at the portion where the caliber of the small intestine changed. (B) An enhanced abdominal CT scan showing a small bowel intussusception with a long tube in the intestinal lumen

**FIGURE 2:**
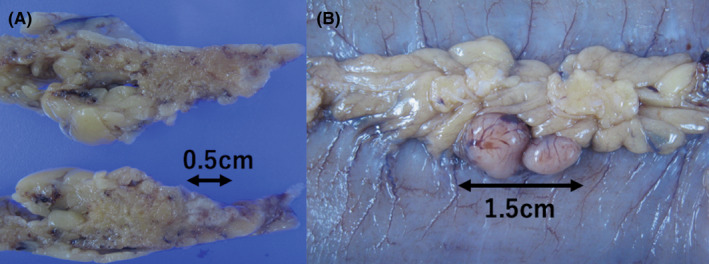
(A) A white nodule of approximately 0.5 cm was found in the resected greater omentum. This was pathologically detected to have chronic inflammatory infiltration with fat necrosis, lymphocytes, and histiocytosis. (B) Two white nodules of approximately 1.5 cm were discovered on the serosal surface of the jejunum. These were pathologically diagnosed as gastrointestinal stromal tumors (GISTs) and judged as very low risk by the Modified Fletcher Classification System

NF1 is a neurocutaneous disorder that causes 25% of all gastrointestinal tract lesions.^1^ This is usually in the form of multiple tumors or plexiform lesions in the gastrointestinal tract and extraintestinal sites.^1^, ^2^ Multiple tumors in the small intestine, particularly the jejunum, are commonly NF1‐associated GISTs.^2^

In this case, the adhesion and cord‐like structure between the greater omentum and mesentery caused the obstruction. The insertion of the long tube may have induced bowel intussusception.^3^ The two GISTs in the jejunum may also have contributed to the intussusception. Thus, the multiple NF1‐associated intra‐abdominal lesions may have affected the onset and hampered treatment of the obstruction. The smaller lesions in the abdomen were not detectable on the abdominal CT, and it is, therefore, important to be aware that small gastrointestinal tumors may complicate the management of patients with NF1.

## CONFLICT OF INTEREST

The authors declare no conflict of interests for this article.

## PATIENT CONSENT FOR PUBLICATION

Obtained.
